# Mesenchymal tumor-induced Fanconi syndrome: A novel classification of Fanconi syndrome

**DOI:** 10.1097/MD.0000000000044845

**Published:** 2025-09-26

**Authors:** Menghua Yuan, Baoping Wang, Hongzhi Li, Jin Cui, Kunling Wang, Fangqiu Zheng, Zhongshu Ma, Qing He, Ming Liu

**Affiliations:** a Department of Endocrinology, General HospitalTianjin Medical University General Hospital, Tianjin, China.

**Keywords:** Fanconi syndrome, fibroblast growth factor 23, mesenchymal tumor, tumor-induced osteomalacia

## Abstract

**Rationale:**

Fanconi syndrome (FS) caused by mesenchymal tumors is rarely reported, and these tumors are usually small and difficult to detect. However, if found and removed, the FS can be cured.

**Patient concerns:**

Five patients were included in this study, and their clinical features, treatments, and prognoses of tumor-induced FS were reviewed and summarized.

**Diagnoses:**

Mesenchymal tumor-induced FS was diagnosed based on laboratory, imaging, and pathological examinations.

**Interventions:**

We performed tumor resection surgery and administered a symptomatic treatment regimen that included neutral phosphorus solution, calcitriol, calcium, and potassium supplementation, and acidosis correction.

**Outcomes:**

Two patients were cured, while 3 other patients showed transient symptomatic improvement postoperatively but still required symptomatic treatment due to tumor recurrence.

**Lessons:**

Our case report and literature review demonstrate that mesenchymal tumors should be included in the spectrum of disorders associated with FS and that FS may represent end-stage tumor-induced osteomalacia (TIO) in certain individuals. The diagnosis of FS requires consideration of tumor-induced causes, with early detection and complete resection being crucial for a cure.

## 1. Introduction

Fanconi syndrome (FS) is a disorder that affects proximal tubular transport and is characterized by the reduced reabsorption of various substances in the kidneys. Clinically, patients present with hypophosphatemia, normoglycemic glycosuria, generalized aminoaciduria, and phosphaturia, which may lead to bone issues such as rickets or osteomalacia. FS can be either hereditary or acquired, and acquired FS typically arises from heavy metal or drug intoxication, immunoglobulin irregularities, autoimmune disorders, or renal disease. Tumor-induced FS is frequently observed in association with hematological malignancies that exhibit paraproteinaemia. Mesenchymal tumors can also cause osteomalacia or rickets by overexpressing fibroblast growth factor 23 (FGF23), which leads to abnormal phosphorus metabolism. This paraneoplastic syndrome is also known as tumor-induced osteomalacia (TIO); however, FS caused by solid tumors,^[1–10]^ especially mesenchymal tumors,^[4–10]^ has rarely been reported. These tumors are usually small and difficult to detect; however, if found and removed, FS can be cured.

This study aimed to retrospectively analyze the clinical manifestations, treatment, and outcomes of 5 such patients treated in our department, with a specific focus on investigating the role of FGF23 in tumor-induced FS pathogenesis through serum measurements and its correlation with disease severity. We also reviewed existing literature to better understand their clinical features.

## 2. Case descriptions

### 2.1. Case 1

A 15-year-old girl was hospitalized for recurrent weakness and walking difficulties with an unknown cause since the age of 9 years. The patient was initially diagnosed with hypophosphatemic rickets without urinary glucose/amino acid testing and received neutral phosphorus solution, calcitriol, calcium, and potassium citrate, which resulted in symptom relief. At 11 years of age, she developed right distal ulnar swelling and pain, with a biopsy-confirmed giant cell tumor. The patient was diagnosed with TIO and underwent tumor resection.

Her postoperative symptoms improved, with alkaline phosphatase (ALP) levels decreasing from 915 U/L to 529 U/L. At 14 years of age, her serum calcium was 1.99 mmol/L, phosphorus 1.05 mmol/L, ALP 843 U/L, 24-hour urine calcium 20.4 mg, phosphorus 1363.2 mg and glucose 2.8 g. She had normal glucose tolerance (NGT). Generalized aminoaciduria was observed, with a tubular phosphorus reabsorption rate of phosphorus (TRP) of 70%, confirming an FS diagnosis. At 15 years of age, she developed sudden right forearm swelling and pain, and her serum phosphorus levels were undetectable. After tumor resection and radiotherapy, her serum phosphorus level rebounded to 0.93 mmol/L post-op. Within 3 months, her serum phosphorus levels dropped to 0.49 mmol/L while ALP rose, which resulted in phosphate therapy reinstatement. Between the ages of 17 and 30 years, the patient had undergone multiple surgeries and radiotherapy for recurrent tumors with persistently low serum phosphorus levels, aminoaciduria, and renal glycosuria.

### 2.2. Case 2

A 57-year-old woman presented with 2-year bone pain and restricted mobility. The laboratory results showed a serum calcium level of 2.2 mmol/L, phosphorus level of 0.5 mmol/L, ALP level of 189U/L, parathyroid hormone (PTH) level of 8.04 pmol/L, and TRP value of 69%. Urinalysis revealed normal glucose and protein levels, mild alanine and aspartate elevations, and other urinary amino acid levels within their respective normal ranges.

She was diagnosed with hypophosphatemic osteomalacia and treated with neutral phosphorus, calcitriol, and calcium tablets, which alleviated bone pain. At 59 years of age, she noticed foot swelling, but dismissed it. Her serum potassium was 3.3 mmol/L, 24-hour urine potassium level was 91 mmol, urine protein level was 236 mg, and urine glucose 4.8 g despite NGT. Urinary amino acid analysis showed generalized aminoaciduria, the patient was diagnosed with FS, and potassium chloride was added.

**Figure 1.**
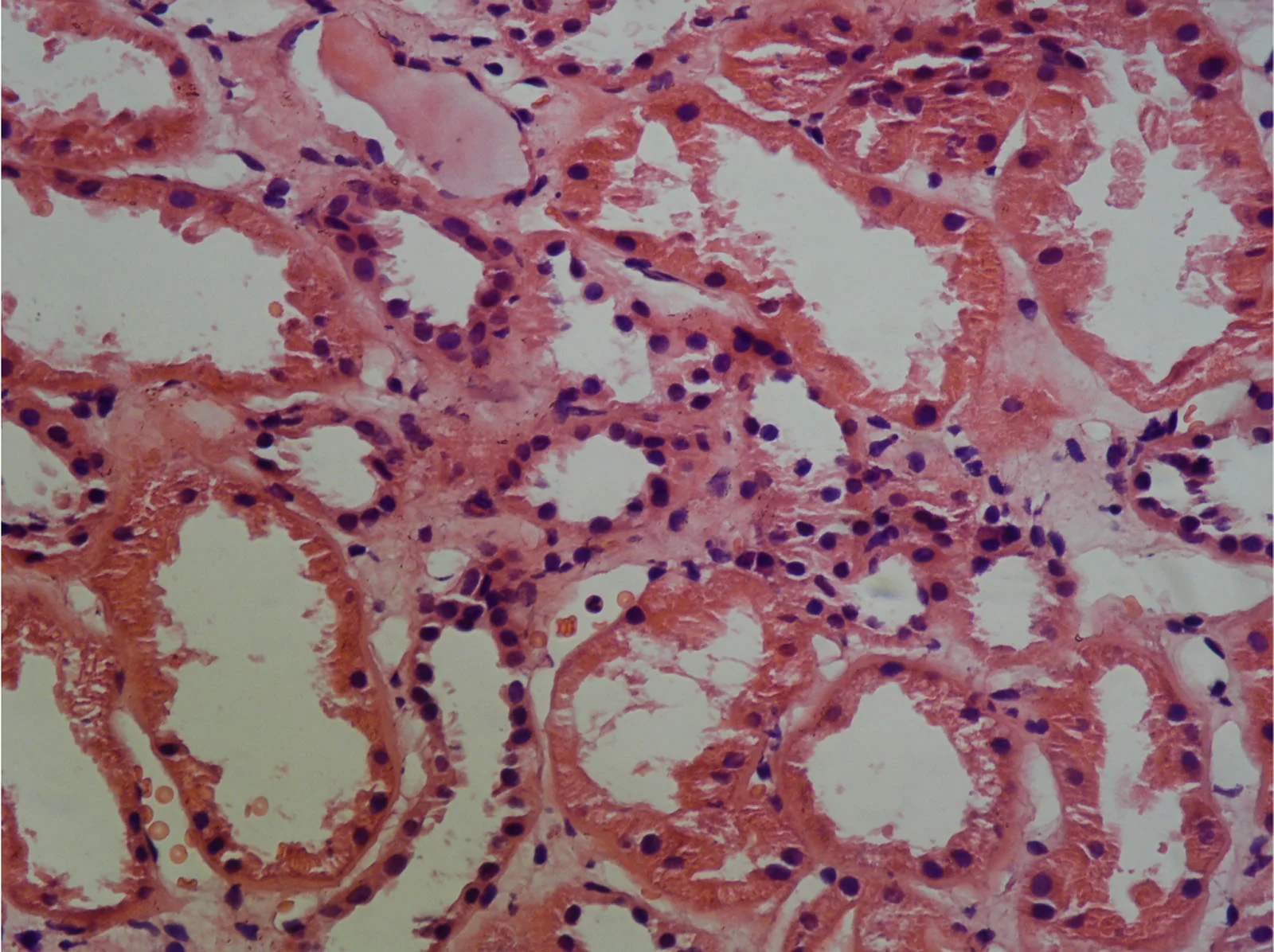
Renal biopsy revealed chronic tubulointerstitial injury. Stain: hematoxylin and eosin.

**Figure 2.**
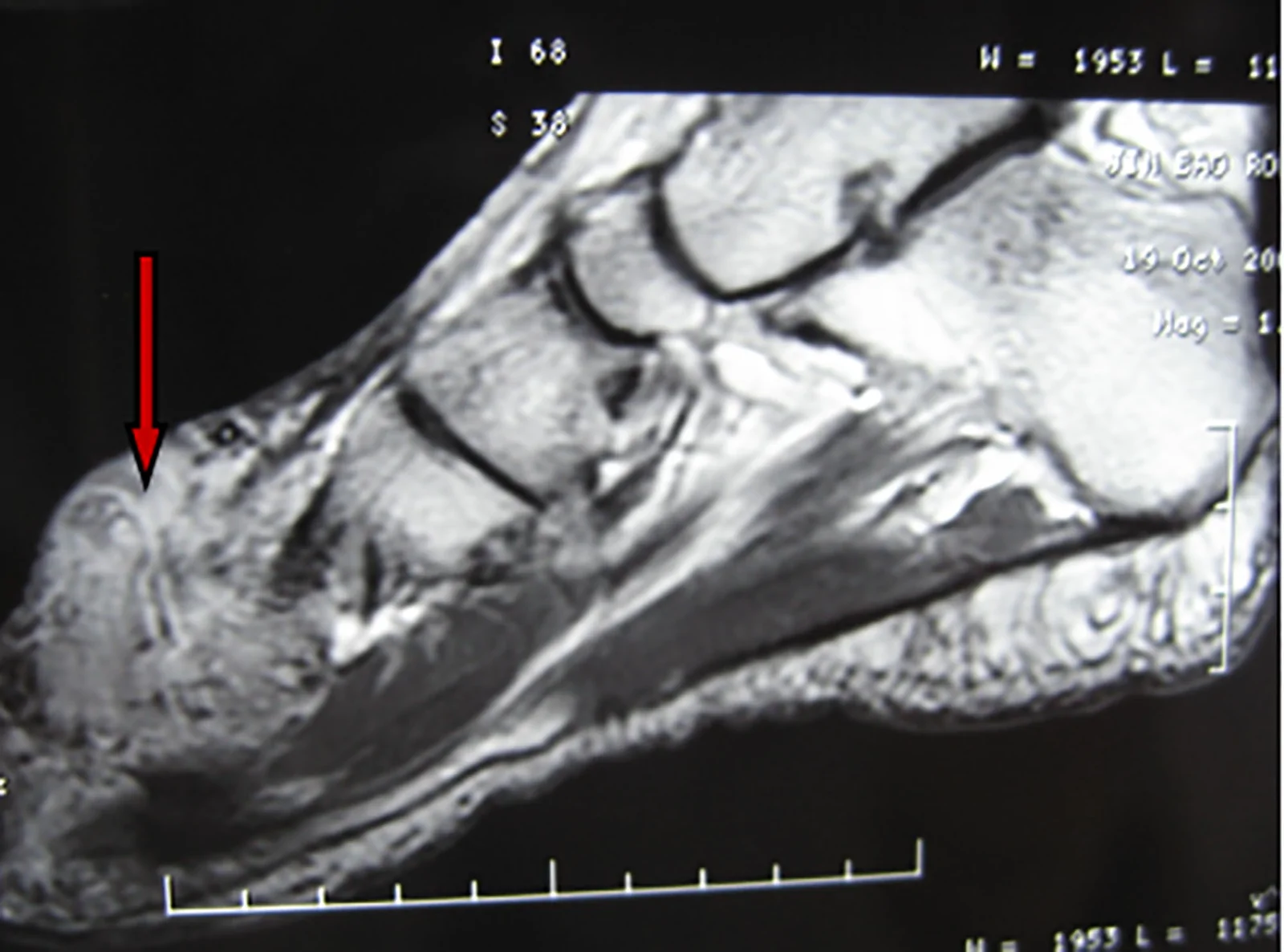
Enhanced magnetic resonance imaging (MRI) of the right foot revealing a soft tissue mass.

**Figure 3.**
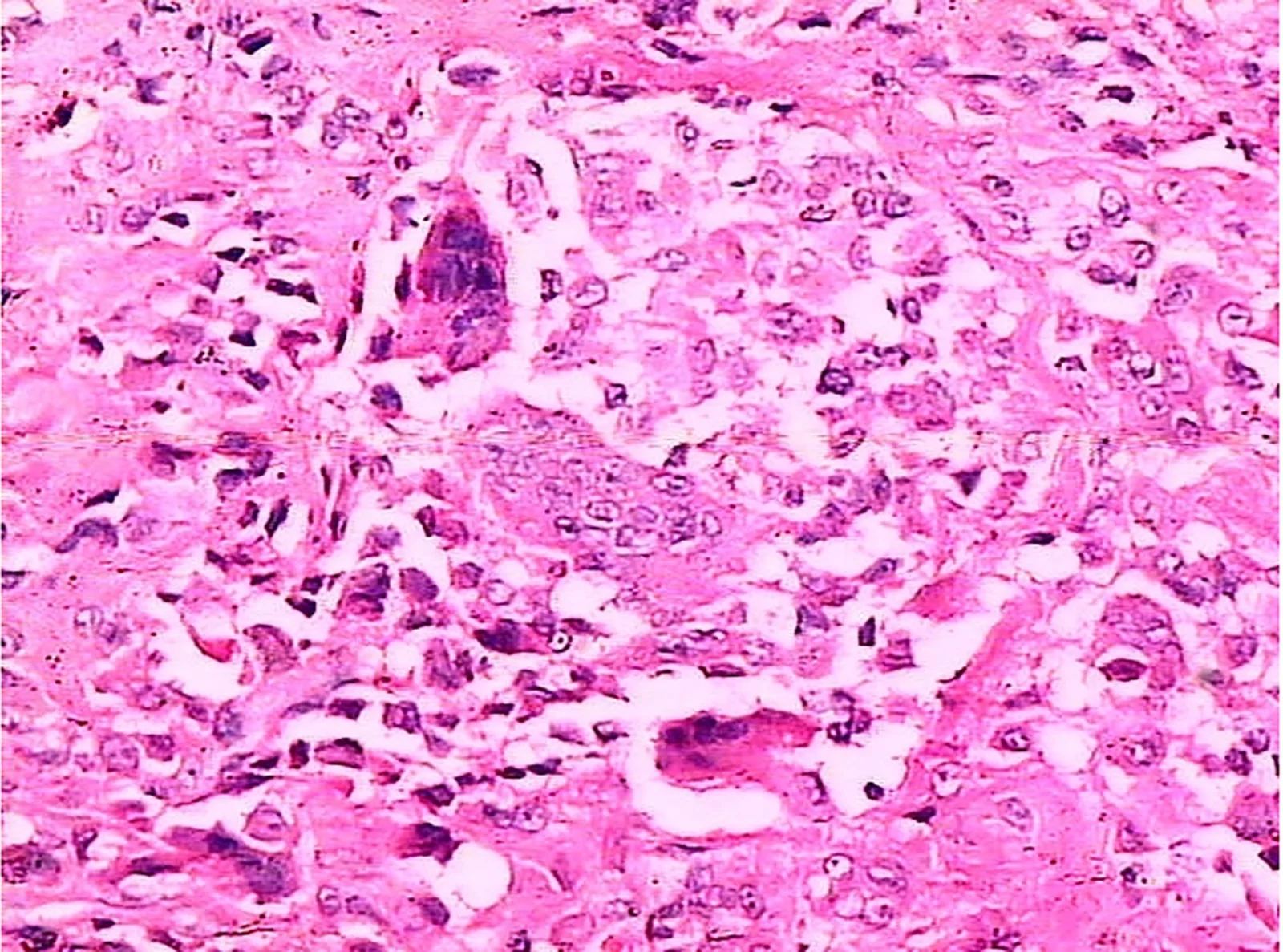
Pathology showing the right foot mass as a giant cell tumor of the tendon sheath. Staining: Hematoxylin and eosin.

At the age of 63 years, deltoid muscle immunofluorescence showed IgA/IgG/C3/FRA deposits along the fascicles, and renal biopsy confirmed chronic tubulointerstitial damage (Fig. 1). Suspecting renal tubular immune injury in FS, prednisone 15 mg/d was attempted but stopped because of unimproved bone pain. Enhanced magnetic resonance imaging (MRI) revealed a mass in the patient’s right foot (Fig. 2), and post-resection pathology confirmed a giant cell tumor of the tendon sheath (Fig. 3). Calcium supplements alone were initially prescribed, with serum calcium 2.40 mmol/L and phosphorus 1.04 mmol/L at 3 days postoperation. At 3 months following the operation, the patient’s serum phosphorus dropped to 0.75 mmol/L, which prompted neutral phosphorus reintroduction. Follow-up MRI at 74 years of age revealed a first metatarsal dorsal mass, which suggested tumor recurrence. The patient refused surgery and was maintained on neutral phosphorus, calcitriol, calcium, and potassium chloride. Subsequent monitoring revealed unrestricted ambulation, with occasional hip discomfort.

### 2.3. Case 3

A 43-year-old woman presented with a decade-long history of bone pain and fatigue. She was diagnosed with FS and renal tubular acidosis at the age of 38 years and was treated with calcitriol, neutral phosphorus, calcium supplements, and sodium bicarbonate. Her symptoms initially improved, but later relapsed and caused a worsening of mobility and kyphosis.

**Figure 4.**
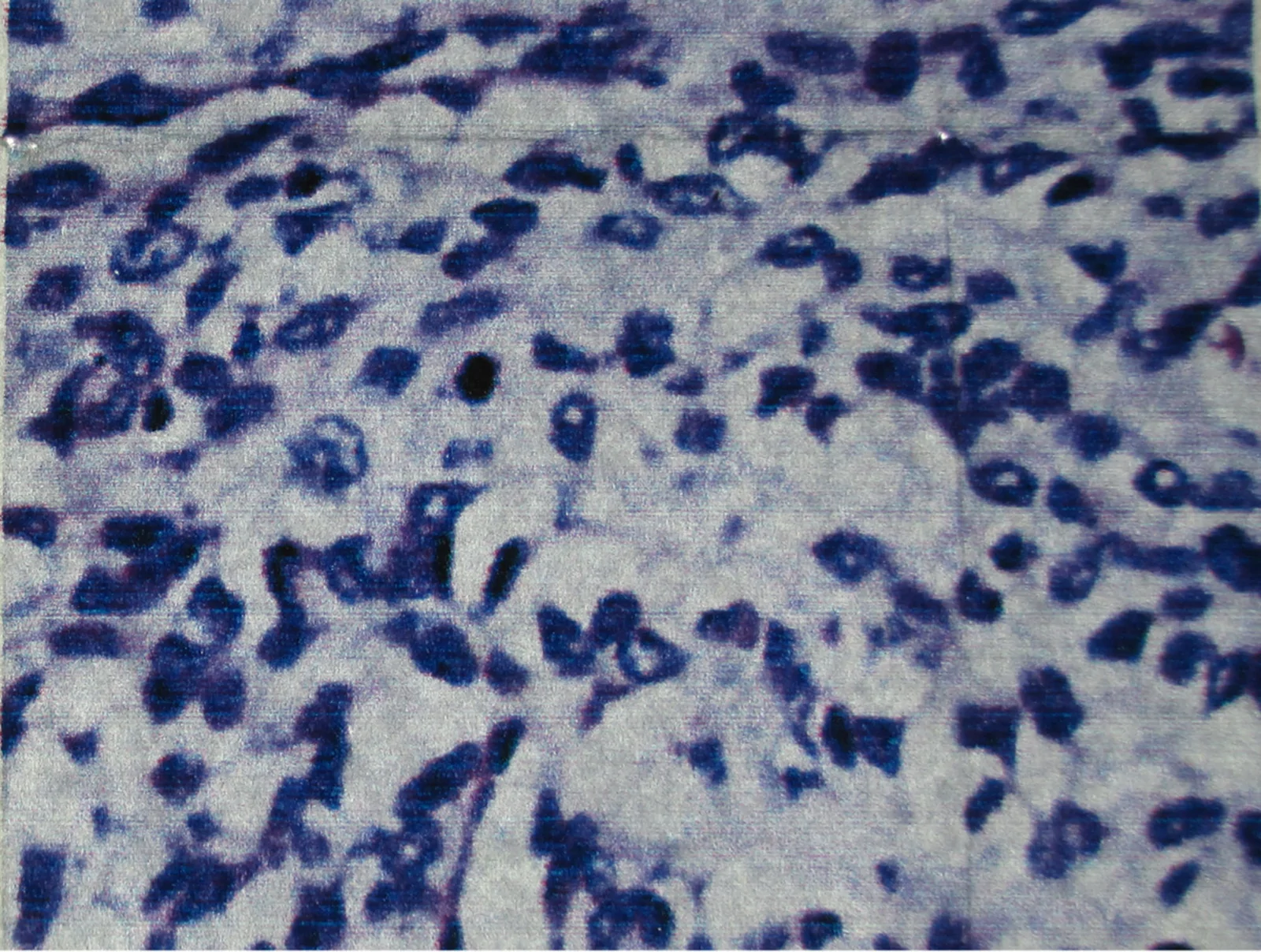
Right ankle mass pathology showing a synovial sarcoma. Staining: Hematoxylin and eosin.

**Figure 5.**
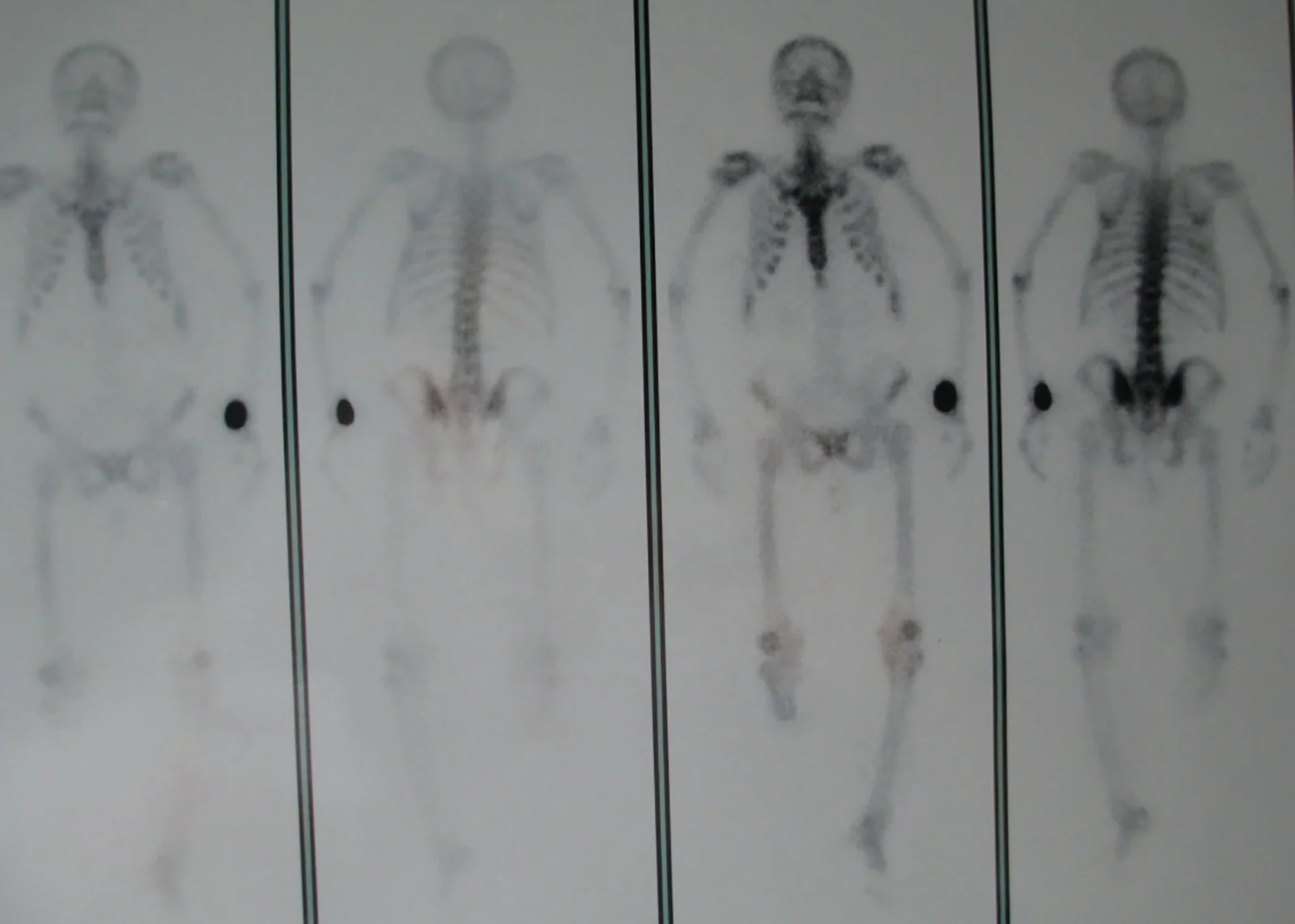
Postoperative bone ECT revealed no evidence of osseous metastasis. ECT = emission computed tomography.

Upon admission, her serum calcium was 2.17 mmol/L, phosphorus was 0.21 mmol/L, PTH was 142 pmol/L, and blood gas was normal. Rheumatoid antibodies were negative, and her 24-hour urine calcium, phosphorus, and glucose levels were 81.6 mg, 531, and 0.36 g with NGT, respectively. Urinary amino acid analysis showed generalized aminoaciduria, and her TRP was 64%. Muscle immunofluorescence revealed striking IgG and FRA deposits that traced the fascicular architecture and prompted clinicians to hypothesize a pathogenic link between FS and renal tubular immune injury. Administration of methylprednisolone 40 mg/d failed to relieve the bone pain after 2 weeks, which led to the discontinuation of corticosteroids. At the age of 46, a mass on the right ankle enlarged with redness, swelling, hardness, and pain was noted. At the age of 51, a right ankle mass biopsy showed synovial sarcoma (Fig. 4), which resulted in below-knee amputation. Post-op bone emission computed tomography showed no metastasis (Fig. 5), the medications were stopped, and 2 weeks later, her serum calcium level was 2.18 mmol/L, phosphorus was 1.18 mmol/L, and ALP was 90 U/L. A year and 5 months postoperatively, her serum phosphorus level was 1.1 mmol/L, 24-hour urine phosphorus was 495 mg, urine sugar was 0.01 g, urine protein was 120 mg, and all urinary amino acids were normal. The patient’s bone pain subsided, and she was able to ambulate with crutches.

### 2.4. Case 4

A 41-year-old woman presented with a 10-year history of tetany and bone pain that had worsened over the prior 9 months. At the age of 34, she developed lower limb deformities and walking difficulties and was diagnosed with undifferentiated spondyloarthritis with ineffective glucocorticoid treatment. She presented thoracic deformities and scoliosis at the age of 38 years and required crutches.

Imaging revealed thoracic curvature, reduced bone density, flattened vertebrae, straightened lumbar lordosis, and femoral head deformities. The bone mineral density *Z* values were −2.9 (L1-L4), −3.7 (femoral neck), and −1.2 (whole body). Her serum calcium level was 2.31 mmol/L, phosphorus was 0.23 mmol/L, potassium was 3.8 mmol/L, ALP was 312 U/L, PTH was 12.20 pmol/L, and 25-hydroxyvitamin D was 27.54 nmol/L. The results of the blood gas analysis, rheumatoid antibody test, and oral glucose tolerance test were normal. The 24-hour urine calcium was 46.4 mg, phosphorus was 440.8 mg (TRP 71%), and sugar was 8.34 g. Urinary amino acid analysis revealed generalized aminoaciduria with significantly elevated glycine and alanine and mildly elevated valine.

**Figure 6.**
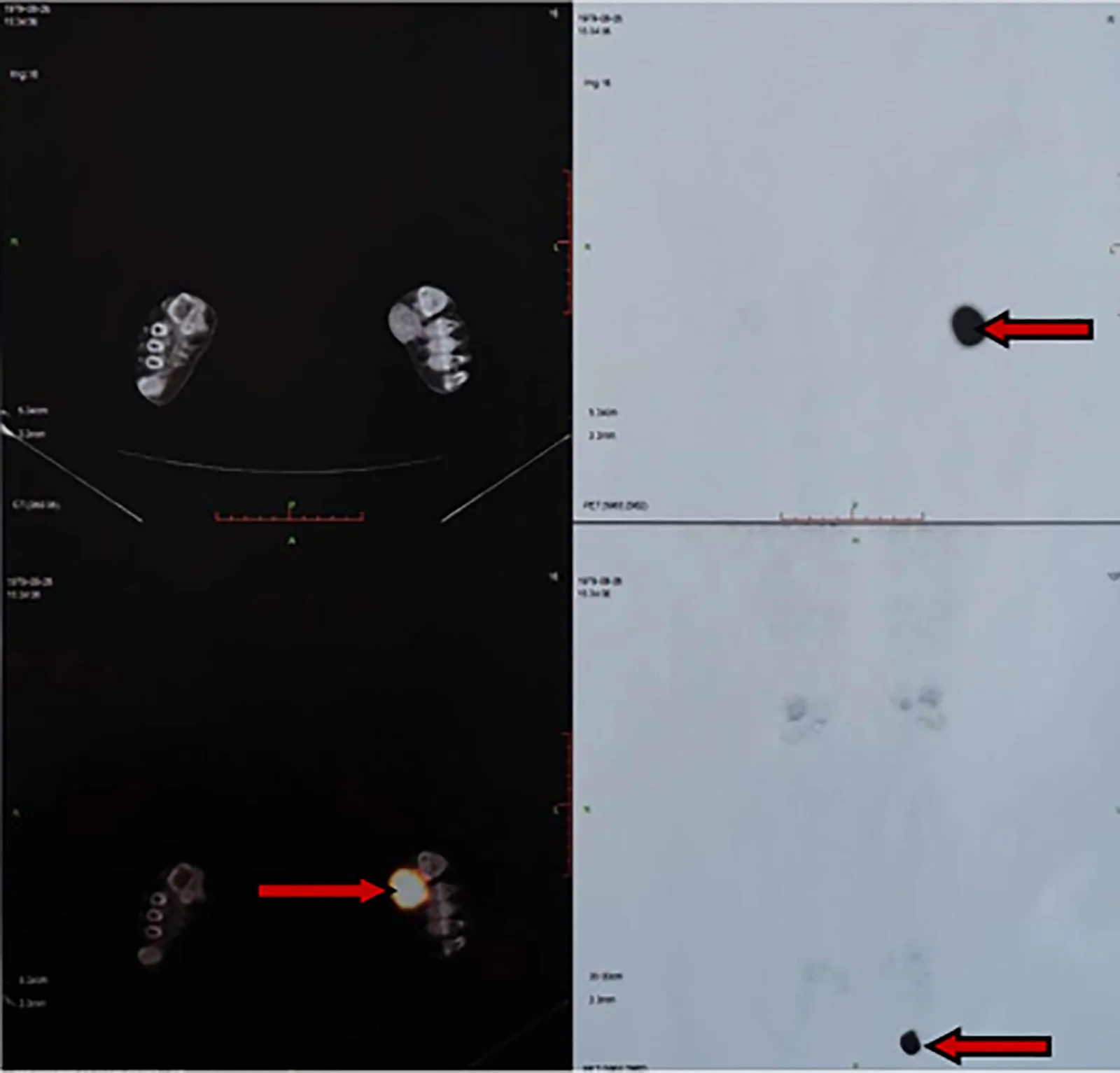
^68^Ga-DOTATATE PET/CT revealed an irregular soft tissue nodule deep in the left plantar first metatarsal, which demonstrated intense DOTATATE uptake.

**Figure 7.**
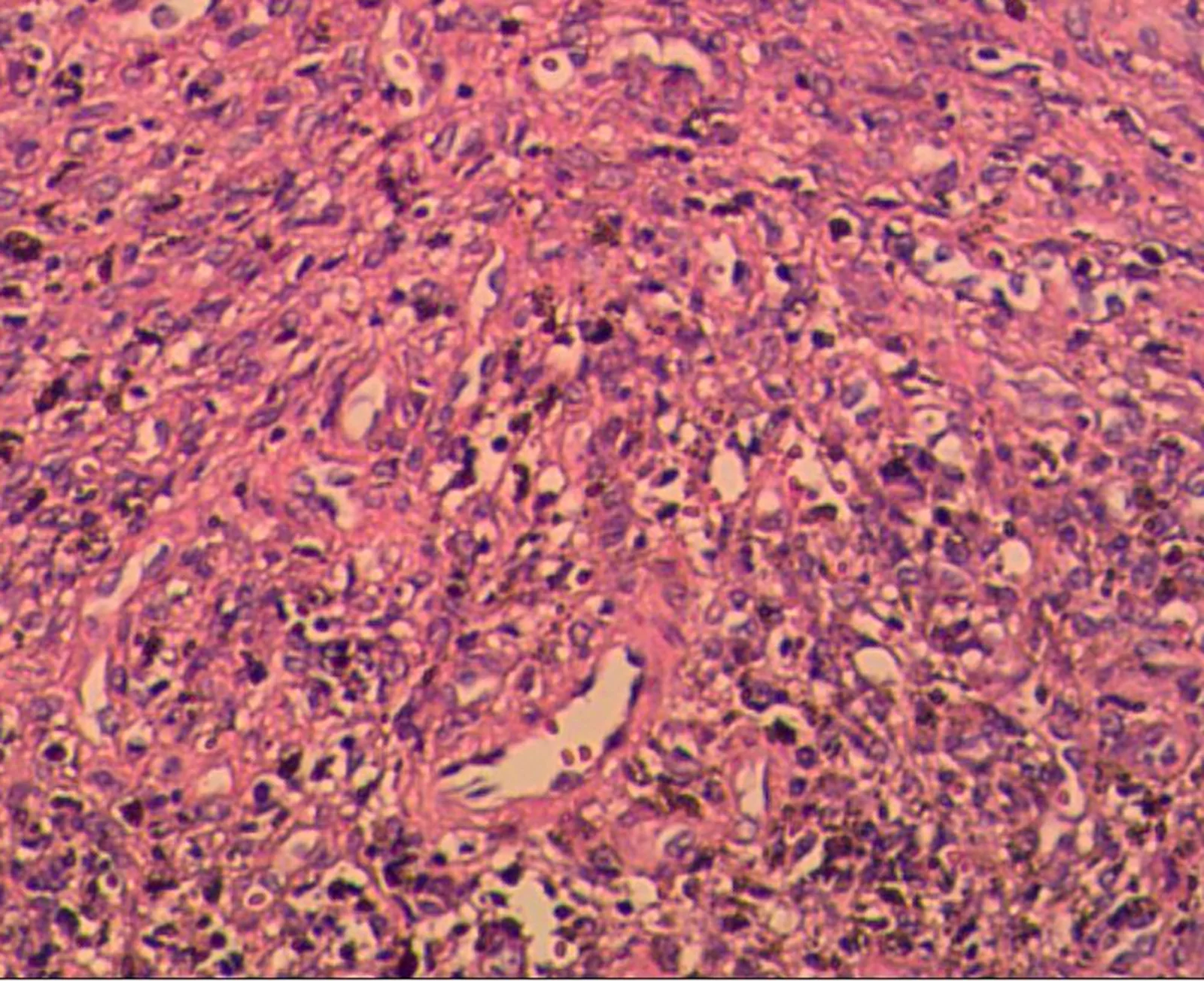
Left plantar mass pathology presented a diffuse-type giant cell tumor. Staining: Hematoxylin and eosin.

The patient was diagnosed with FS and underwent ^18^F-FDG PET/CT, ^68^Ga-DOTATATE PET/CT (Fig. 6), and enhanced MRI, which revealed a tumor around the left first metatarsal bone base. The patient underwent surgery, and pathological examination confirmed a diffuse giant cell tumor (Fig. 7). Immunohistochemistry revealed that CD68+ giant cells and CD34+ vasculature were negative for S-100, SMA, HMB45, melan-A, and SOX-10, with a Ki-67 index of approximately 10%. Treatment with calcium and calcitriol was initiated after the surgery.

One week postoperatively, the serum calcium level was 2.19 mmol/L, phosphorus was 0.73 mmol/L, and 24-hour urinary phosphorus was 438.6 mg (TRP 92%). Six months postoperatively, the patient’s bone pain symptoms resolved, and her walking ability was restored. Follow-up tests showed a serum calcium level of 2.29 mmol/L, phosphorus level of 0.66 mmol/L, 24-hour urinary phosphorus level of 510.2 mg (TRP 86%), urinary sugar level of 0.08 g, and mild increases in urinary glycine and alanine, with normal urinary valine levels. The MRI findings did not rule out residual or recurrent tumors, and neutral phosphorus was added to her treatments.

### 2.5. Case 5

A 43-year-old man presented with 3 years of progressive lower limb weakness and pain. At the age of 40 years, he developed leg-lifting difficulty with “duck gait,” which progressed to mobility impairment and intercostal pain. An evaluation showed normal electromyographic findings and no neurological nor muscular abnormalities. The laboratory tests revealed hypocalcemia (2.09 mmol/L), hypophosphatemia (0.56 mmol/L), elevated ALP (317 U/L), and FGF23 (317 pg/mL, reference range: 23.3–95.4). Urinalysis revealed 24-hour urinary calcium excretion of 144 mg, urinary phosphorus excretion of 734 mg (TRP, 76.7%), abnormal glucose excretion (2.12 g) under NGT, and generalized aminoaciduria.

**Figure 8.**
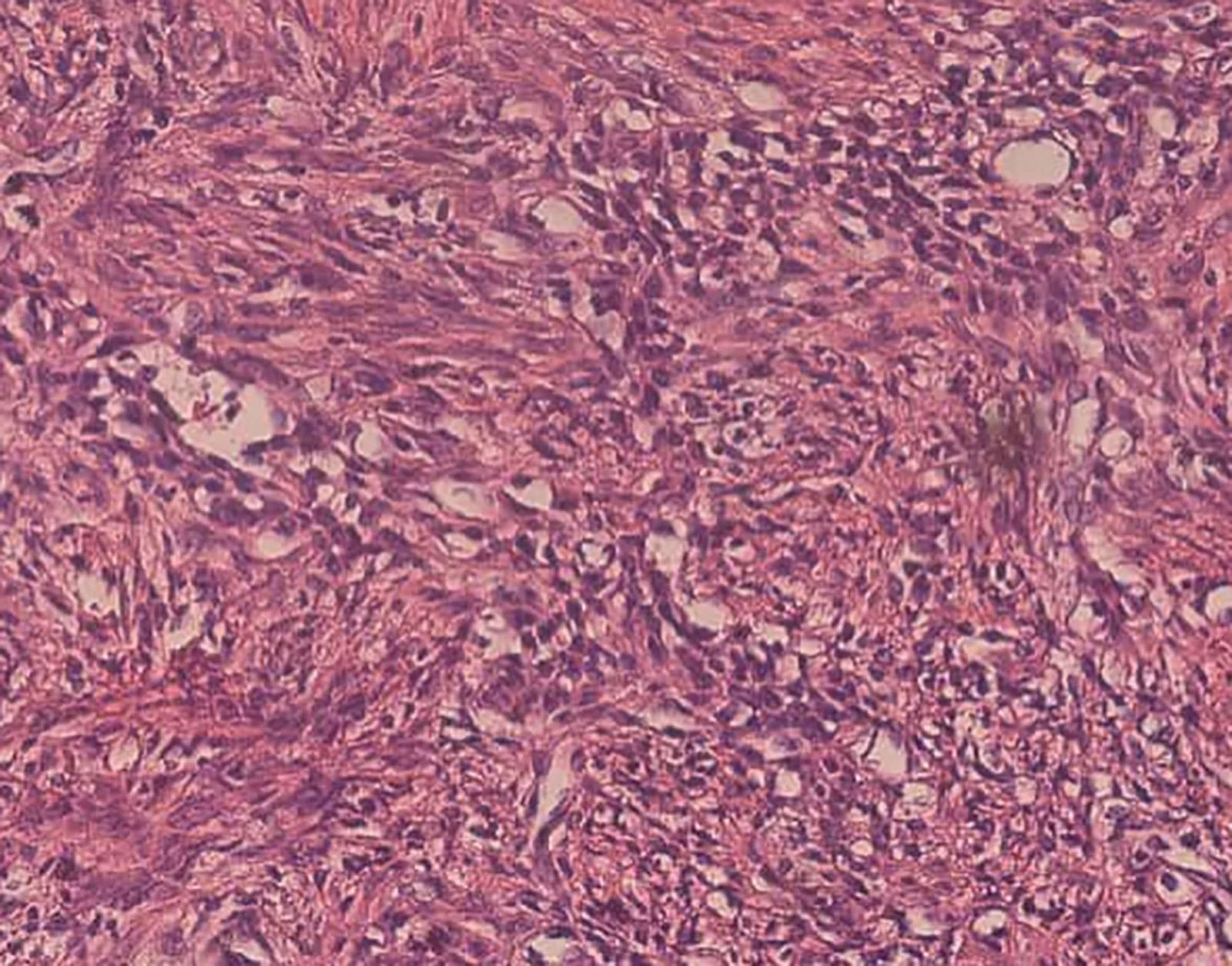
Pathological examination of the right mandibular mass indicated fibrous histiocytoma-like hyperplasia. Staining: Hematoxylin and eosin.

^18^F-FDG PET/CT revealed a hypermetabolic nodule in the right mandible, whereas ^68^Ga-DOTATATE PET/CT revealed abnormal uptake, which was suggestive of a neuroendocrine tumor. CT revealed an 18*13 mm irregular low-density protrusion with patchy high-density areas at the right molar alveolar ridge. MRI confirmed an 18*13 mm contrast-enhancing lesion that compressed the adjacent tissues. The patient underwent a resection of the mandibular lesion, and the pathological results indicated fibrous histiocytoma-like hyperplasia (Fig. 8). Immunohistochemistry revealed scattered CD68 + tumor cells with desmin, SMA, S-100, CD34 negativity, and Ki-67 expression of approximately 5%. One week postoperation, his serum calcium level was 2.02 mmol/L, phosphorus was 1.17 mmol/L, 24h urine calcium was 79.6 mg, phosphorus was 645.7 mg (TRP 93%), and urinary amino acids were normal. Calcium and calcitriol were then administered. At the 1-year follow-up, his serum phosphorus level was 1.4 mmol/L, calcium was 2.46 mmol/L, and urine glucose was negative. The patient achieved resolution of his bone pain and maintained mobility.

Table 1 summarizes the key biochemical and treatment-related data for all 5 cases.

**Table 1:** Clinical data, biochemical markers, and treatments of the 5 patients.

Case	Age/sex	Serum calcium (mmol/L)	Serum phosphorus (mmol/L)	24h urinary phosphorus (mg)	FEp (%)	TRP (%)	TmP/GFR (mmol/L)	24h urinary glucose (g)	AA	Elementary phosphorus supplementation	Calcitriol supplementation	Calcium and vitamin D supplementation
1	15/F	1.99 (low)	1.05→0.49 (low)	1363.2 (high)	30	70	0.98	2.8	+	Neutral-P	Yes	Yes
2	57/F	2.2	0.5 (low)	623.1	31	69	0.47	4.8	+	Neutral-P	Yes	Yes
3	43/F	2.17	0.21 (severe low)	531	36	64	0.19	0.36	+	Neutral-P	Yes	Yes
4	41/F	2.31	0.23 (severe low)	440.8	29	71	0.22	8.34	+	Neutral-P	Yes	Yes
5	43/M	2.09 (low)	0.56 (low)	734	23.3	76.7	0.53	2.12	+	No	Yes	Yes

AA = aminoaciduria, FEp = fractional excretion of phosphate, Neutral-P = neutral phosphorus solution, Suppl. = supplementation, TmP/GFR = tubular maximum reabsorption rate of phosphate/glomerular filtration rate, TRP = tubular reabsorption of phosphate.

## 3. Discussion

FS was first described by Fanconi et al in 1931; however, the pathogenesis of this condition remains unclear. FS caused by tumors is commonly observed in malignant hematological neoplasms, such as multiple myeloma. This is believed to result from immunoglobulin light-chain deposition in the renal tubules that form an electron-dense material and impair cell function. Notably, FS caused by mesenchymal tumors has rarely been reported.^[4–10]^

In this study, 5 patients presented with symptoms and were diagnosed with hypophosphatemia, and mesenchymal tumors were later identified. This initially suggested the presence of small, elusive tumors consistent with TIO. In patient 1, hypophosphatemia was detected without concurrent urine glucose/amino acid measurements, followed by tumor identification and FS diagnosis. Post-resection showed marked ALP reduction and serum phosphorus elevation with clinical improvement, which suggests tumor causality. However, tumor recurrence prevented serum phosphorus normalization by causing renal tubular dysfunction, which was evidenced by persistent aminoaciduria and normoglycemic glycosuria.

In Case 2, the patient initially presented with hypophosphatemic osteomalacia (excluding FS due to normal urine glucose, protein, and glycine levels). After 2 years, normoglycemic glycosuria, generalized aminoaciduria, and microalbuminuria emerged, which confirmed the diagnosis of FS. Other causes such as heavy metal toxicity and autoimmune disorders were excluded, although foot masses were initially missed. A renal biopsy suggested immune-mediated tubular injury, which prompted glucocorticoid therapy, although the treatment resulted in no clinical improvement.

Similar to patient 1, foot tumor resection temporarily normalized the serum phosphorus levels; however, hypophosphatemia recurred 3 months postoperation due to incomplete resection, which was confirmed by tumor recurrence a decade later. The case of patient 2 reveals that hypophosphatemic osteomalacia and FS can occur sequentially in the same patient. Elevated urinary phosphorus levels indicate proximal tubular dysfunction, whereas the FS reflects broader proximal tubular impairment. We therefore inferred that the FS might represent end-stage TIO in certain patients.

In case 3, the patient underwent amputation for malignant pathology. Postoperative bone emission computed tomography revealed no metastases, thus confirming complete resection. Serum phosphorus and urinary glucose/amino acids normalized after 15 months, which confirmed the tumor-induced FS etiology. Mesenchymal tumors should also be included in the FS spectrum, and we recommend a “tumor-induced FS” classification to distinguish such tumors from other causes. The last 2 cases involved recent hospitalizations, thus highlighting the accuracy of ^68^Ga-DOTATATE PET/CT for rapid diagnosis and tumor localization. Following resection, serum phosphorus in patient 4 transiently elevated and then declined, mirroring the pattern seen in patients 1 and 2. In patient 5, serum phosphorus and urinary amino acids normalized postoperatively and remained stable at 14 months due to complete lesion resection without recurrence.

The tumors in this series involved the limbs and skull, affecting both bone and soft tissue with ill-defined borders. Histologically, they are characterized by an infiltrative growth pattern composed of morphologically heterogeneous mesenchymal cells that lack a specific architectural arrangement. Furthermore, pathological reports include giant cell tumors of the bone and tendon sheath, synovial sarcoma, diffuse giant cell tumors, and fibrous histiocytoma-like proliferation. In combination with the clinical presentation, biochemical characteristics, and special endocrine changes, all lesions met the clinical and pathological features of phosphaturic mesenchymal tumors.^[11]^

How do mesenchymal tumors trigger FS? In patients 2 and 3, glucocorticoid immunosuppressive therapy was ineffective, which indicates that tumor-induced FS pathogenesis differs from that of autoimmune diseases or paraproteinemia. The latter 2 typically respond to glucocorticoids. Renal tubular phosphate reabsorption relies on the sodium-phosphate (Na/Pi) cotransporter. Reduced expression of type 2 Na/Pi cotransporter (NaPi-2) in the proximal tubule may lower the renal phosphate threshold in patients with TIO. Notably, Israeli researchers have found siblings in whom NaPi-2a mutations were linked to FS, thus suggesting that the transporter has a direct role in FS.^[12]^ Furthermore, impaired NaPi-2a expression may disrupt other proximal tubule transporters via unknown mechanisms, thereby causing broader proximal tubular dysfunction.

Current evidence indicates that TIO results from tumor-derived phosphaturic factors that include FGF23, matrix extracellular phosphoglycoprotein (MEPE), and secreted frizzled-related protein 4 (sFRP4).^[13,14]^ FGF23 is a 251-amino acid hormone that is predominantly expressed in osteoblasts and osteocytes, although it can also be ectopically detected in tissues such as the liver, brain, heart, thyroid, intestine, and skeletal muscle under pathological conditions. Its secretion is physiologically stimulated by phosphate, PTH, inflammatory cytokines, and 1,25-dihydroxyvitamin D3 (1,25(OH)2D3). Functionally, FGF23 decreases the renal threshold for phosphate reabsorption by downregulating the expression of sodium-phosphate cotransporters, including NaPi-2a, NaPi-2c, and Pit-2, in the proximal tubule.^[15]^ Additionally, it inhibits 1-alpha hydroxylase activity, thereby impairing the conversion of 25-hydroxyvitamin D (25OHD) to its active form, 1,25(OH)2D3.

Our study utilized FGF23 measurements to elucidate its role in mesenchymal tumor-induced FS. As a key phosphaturic hormone, FGF23 showed significant elevation in patient 5 (317 pg/mL vs normal 23.3–95.4 pg/mL), which confirms its involvement in the renal phosphate wasting characteristic of FS. This finding supports the hypothesis of Norden et al that tumor-secreted FGF23 may underlie FS in TIO.^[8]^ Unfortunately, owing to past laboratory limitations, only patient 5 was tested for serum FGF23. Notably, FGF23 measurements provide important insights into disease mechanisms for the following 3 reasons: elevated FGF23 levels correlate with severe hypophosphatemia and low TRP, may explain the progression from isolated phosphaturia (TIO) to generalized proximal tubulopathy (FS), and may serve as a potential biomarker for tumor recurrence monitoring. We propose that the long-term exposure of the proximal tubule to certain factors may progress from isolated functional impairment to extensive dysfunction, thereby causing FS. But why do only a few mesenchymal tumors induce FS? Jiang et al found that SLC34A3 and XPR1 polymorphisms correlated with TIO and FS,^[10]^ thus suggesting new research directions for tumor-induced FS pathogenesis through FGF23 and related genetic polymorphisms.

## 4. Conclusion

This case series establishes mesenchymal tumors as a clinically significant etiology of acquired FS and proposes a novel subclassification of tumor-induced FS. Our findings reveal: pathogenetic links – mesenchymal tumors (e.g., giant cell tumors, synovial sarcoma) can induce FS through FGF23-mediated phosphaturia and proximal tubular dysfunction, with FS potentially representing end-stage TIO; diagnostic challenges—the tumors are often small, anatomically occult (e.g., in extremities/jaws), and may evade initial detection, thereby leading to delayed diagnosis (mean delay: 5–10 years in our cohort); critical intervention – complete surgical resection is curative, as evidenced by normalized serum phosphorus, TRP, and urinary solute excretion in patients 3 and 5. Incomplete resection (patients 1, 2, and 4) resulted in recurrence and persistent FS; therapeutic implications – glucocorticoids are ineffective, which underscores the distinct pathophysiology of autoimmune/paraproteinemia-associated FS; and clinical recommendation – FS warrants systematic screening for mesenchymal tumors using functional imaging (e.g., ^68^Ga-DOTATATE PET/CT), with early resection essential for cure.

While our research underscores the importance of recognizing mesenchymal tumor-induced FS as a curable cause of acquired phosphate wasting, our study has some limitations that should be considered when interpreting our findings. Notably there have been inconsistent historical measurements of FGF23, which was only definitively tested in 1 patient in this study. To build on these findings, future studies must delve into the potential genetic mechanisms, such as SLC34A3 or XPR1 polymorphisms, which may contribute to the development of FS. Furthermore, prospective studies are urgently needed to establish optimal surveillance protocols for monitoring tumor recurrence. Ultimately, in clinical practice, it is essential to rule out this underlying mesenchymal tumor in all patients with acquired FS, as its identification offers a path to a complete cure, especially when detected early.

## Acknowledgments

We extend our sincere gratitude to the patients and their families for their participation. We are also indebted to Dr Zhu and Dr Wang for their expert preparation and interpretation of the pathological specimens. We would like to thank Editage (www.editage.cn) for English language editing.

## Author contributions

**Conceptualization:** Menghua Yuan, Qing He, Ming Liu.

**Data curation:** Baoping Wang, Hongzhi Li, Jin Cui, Kunling Wang, Fangqiu Zheng, Zhongshu Ma.

**Supervision:** Qing He, Ming Liu.

**Writing – original draft:** Menghua Yuan.

**Writing – review & editing:** Qing He, Ming Liu.
